# Putting Ostracism into Perspective: Young Children Tell More Mentalistic Stories after Exclusion, But Not When Anxious

**DOI:** 10.3389/fpsyg.2016.01926

**Published:** 2016-12-22

**Authors:** Lars O. White, Annette M. Klein, Kai von Klitzing, Alice Graneist, Yvonne Otto, Jonathan Hill, Harriet Over, Peter Fonagy, Michael J. Crowley

**Affiliations:** ^1^Department of Child and Adolescent Psychiatry, Psychotherapy and Psychosomatics, University of LeipzigLeipzig, Germany; ^2^Institute of Psychology, Goethe University FrankfurtFrankfurt, Germany; ^3^School of Psychology and Clinical Language Sciences, University of ReadingReading, UK; ^4^Department of Psychology, University of YorkYork, UK; ^5^Research Department of Clinical, Educational and Health Psychology, University College LondonLondon, UK; ^6^Yale Child Study Center, Yale University, New HavenCT, USA

**Keywords:** social exclusion, early childhood, theory of mind, mentalizing, prosocial behavior

## Abstract

Much is known about when children acquire an understanding of mental states, but few, if any, experiments identify social contexts in which children tend to use this capacity and dispositions that influence its usage. Social exclusion is a common situation that compels us to reconnect with new parties, which may crucially involve attending to those parties’ mental states. Across two studies, this line of inquiry was extended to typically developing preschoolers (Study 1) and young children with and without anxiety disorder (AD) (Study 2). Children played the virtual game of toss “Cyberball” ostensibly over the Internet with two peers who first played fair (inclusion), but eventually threw very few balls to the child (exclusion). Before and after Cyberball, children in both studies completed stories about peer-scenarios. For Study 1, 36 typically developing 5-year-olds were randomly assigned to regular exclusion (for no apparent reason) or accidental exclusion (due to an alleged computer malfunction). Compared to accidental exclusion, regular exclusion led children to portray story-characters more strongly as intentional agents (intentionality), with use of more mental state language (MSL), and more between-character affiliation in post-Cyberball stories. For Study 2, 20 clinically referred 4 to 8-year-olds with AD and 15 age- and gender-matched non-anxious controls completed stories before and after regular exclusion. While we replicated the post regular-exclusion increase of intentional and MSL portrayals of story-characters among non-anxious controls, anxious children exhibited a decline on both dimensions after regular exclusion. We conclude that exclusion typically induces young children to mentalize, enabling more effective reconnection with others. However, excessive anxiety may impair controlled mentalizing, which may, in turn, hamper effective reconnection with others after exclusion.

## Introduction

The preschool years have long been noted for fundamental advances in mentalizing – the social-cognitive capacity to construe oneself and others in terms of intentional mental states (Dennett, 1978; Fonagy et al., 2002). The timetable of the development of mentalizing has received much attention over the past decades (see Wellman, 2014). Yet, as mentalizing enters the child’s repertoire, the question arises as to when and which children make use of this new mental tool by mentalizing in varying social contexts. Despite the importance of such work for theories of mentalizing – particularly the interaction of mentalizing with motivational states and stress regulation (Ickes and Simpson, 2001; Tomasello et al., 2005; Fonagy and Luyten, 2009) – few if any experimental studies directly address the roles of context and disposition in mentalizing. Indeed, if mentalizing varies systematically as a function of context or arousal, it could be crucial to assess context-specific mentalizing capacities of clinical populations whose symptoms primarily appear under certain conditions, such as anxiety disorder (AD).

Mentalizing may be relevant to a broad set of social interactions, from dyadic emotion regulation and caregiving to cooperative and competitive interactions, more broadly (Dennett, 1987; Moore and Frye, 1991; Fonagy et al., 2002). Accordingly, individuals may be thought to mentalize in a wide variety of contexts with many authors proposing that mentalizing permeates our everyday social cognition (e.g., Wellman, 2014). Importantly, for the present purposes, the degree and cognitive control of mentalizing may still show cross-situational variation as the need and expectation to cooperate and compete with others fluctuates.

With this in mind, one important context for inducing shifts in social cognition may be exclusion from groups. As a fundamental process for humans, social exclusion blocks access to various group resources that, across phylogeny, were essential to survival, from group protection, to collaboration for provisions, to exchange of social information (Leary and Cottrell, 2013). Potentially for this reason, threats of exclusion still act as powerful triggers for conformity. Serving as a deterrent for exploiting others, threats of exclusion therefore also stabilize and promote cooperation (Ouwerkerk et al., 2005; Williams, 2009; Feinberg et al., 2014). Critically, to act on the first hints of and avoid further exclusion, excluded parties may potentially increase vigilance regarding social cues to promote more skillful re-affiliation (Pickett and Gardner, 2005; see below). Yet, few studies address such exclusion-responses early in development, especially with young children.

To date, the bulk of work on peer exclusion in early childhood has focused on risk factors for chronic peer rejection and its adverse developmental sequelae (e.g., Crick et al., 1999; von Klitzing et al., 2014). Consequently, we know relatively little about typical and atypical responses to experimental social exclusion at this age. A handful of studies examining exclusion among preschoolers uses indirect primes where the child observes the exclusion of a third party. Even this simple manipulation leads some preschoolers to behave in a way that suggests a reconnection motive has been engaged, including more accurate imitation of others (Over and Carpenter, 2009b; Watson-Jones et al., 2014) and drawing pictures of themselves and friends standing closer to one another (Song et al., 2015). Consistent with these findings, a recent study exposed preschoolers to firsthand exclusion while playing the virtual ball-toss game, Cyberball, also finding increased fidelity of imitation post-exclusion (Watson-Jones et al., 2016). Overall, these findings in young children resemble research on adults, showing increased affiliative tendencies (e.g., conformity, generosity, mimicry) following exclusion compared to control conditions (see Molden and Maner, 2013).

Given the behavioral affiliation-inducing effect of social exclusion, we sought to examine whether young children would also attend to mental states more closely after exclusion. Indeed, some theorists propose that exclusion gives rise to a state of “social hunger” (Gardner et al., 2000, p. 486) that stimulates social monitoring processes, akin to increased attention to food stimuli after fasting. Among adults, social exclusion thus promotes attentional biases to relevant social information (Pickett and Gardner, 2005), including others’ perspectives (Knowles, 2014). Coping with social exclusion by attending to other’s perspectives and mental states may enable more adept detection and selection of new partners likely to reciprocate while weeding out less promising partners. Many affiliative actions (e.g., helping) could also improve (in quality and quantity) if excluded parties attend to mental states of potential targets for re-affiliation so as to tailor affiliative actions to the needs, goals, and knowledge of those targets (Tomasello et al., 2005). Despite its clear potential for informing developmental theories on mentalizing, little or no work currently extends this work to social exclusion in young children. We therefore sought to address this gap in the literature with Study 1.

In a second Study, we moved beyond examining mentalizing in typically developing youth, to consider young children with elevated anxiety concerns. Deficits in social cognition and mentalizing have been linked to numerous childhood psychopathologies (Sharp et al., 2008). However, in the case of AD, one of the most prevalent conditions in childhood (Costello et al., 2011), the deficit in mentalizing has proven somewhat difficult to pin down (see Banerjee, 2008). While socially anxious young children have shown normal responses on standard false-belief tasks in most studies (Banerjee and Henderson, 2001; Broeren et al., 2013; but see Colonnesi et al., 2016), they have exhibited impairments in social behaviors requiring insight into mental states, in self-presentational tactics toward peers as well as in understanding the causes and emotional effects of unintentional insults (Banerjee and Watling, 2010).

Arguably, this pattern of data could be at least partly accounted for by context-specific deficits in mentalizing under affectively charged conditions, such as social exclusion. Thus, it has been proposed that controlled mentalizing varies as a function of the arousal induced by a specific context, following a trajectory of an inverted u-curve, i.e., first rising and then falling with increasing arousal (Fonagy and Luyten, 2009). Given the excessive negative arousal inherent in acute anxiety, deficits in stress-related mentalizing may typify anxious children (Nolte et al., 2011), much like what has been shown by pilot data in adults with panic disorder (Rudden et al., 2006). Moreover, in acute anxiety, one’s own and others’ thoughts often take on an imminent and threatening quality, which may derive from insufficient distinctions between one’s mental representation and reality, one of the hallmarks of a prementalizing mode (e.g., fear of imagined catastrophic separation outcomes, fear of negative evaluation by others; Fonagy et al., 2002). Thus, in Study 2 we examine young anxious children’s usage of mentalizing in an acute stress-context, following social exclusion.

In the current pair of studies, we used the virtual ball-toss game “Cyberball” (Williams et al., 2000) to manipulate social exclusion. Children were ostensibly connected to the Internet to toss a ball back and forth with two peers. The peers eventually stopped passing the ball to the subject (exclusion). Initially, we demonstrated that 5-year-olds excluded in Cyberball report higher threat to relational needs and attribute more bad intentions to co-players on post-Cyberball puppet interviews, as well as more tattling to experimenters on co-players than included children (White et al., unpublished).

Here, to capture young children’s mentalizing and affiliative responses to exclusion, we adapted a widely used narrative story-stem task that children completed before and after Cyberball. In this task, children are exposed to scripted story-beginnings and asked to show and tell the experimenter what happens next using toy figures (see Emde et al., 2003). Story-completion measures have a long history of use in studies of typical and atypical child development. Many of these studies have focused on the way children portray characters in their stories (e.g., parents, children) as a window to their internal representations of themselves and others (see Yuval-Adler and Oppenheim, 2014 for a review). Accordingly, studies suggest that the manner in which children portray the child- and parent-characters in their stories partly overlaps with actual real-world behaviors of these children and their caregiving experiences (Oppenheim et al., 1997; Toth et al., 1997). For example, the magnitude of children’s affiliative and aggressive themes in such narratives is associated with the tendency to express similar behaviors in various social contexts, as reported by clinicians, parents, or teachers (e.g., Kochanska et al., 1996; Hill et al., 2007; von Klitzing et al., 2007).

Recently, the story-stem approach has been broadened to assess children’s tendency to mentalize in their stories (Hill et al., 2007, 2008; Luyten and Fonagy, 2014). More specifically, this approach assesses the degree to which children treat story-characters as intentional agents, i.e., portraying figures *as if* they have goals and mental states.^1^ For story-stems with positive themes, previous research has documented an association between mentalizing, as indexed by the story-stem approach, and theory of mind, as indexed by a traditional false-belief measure (Hill et al., 2008). By contrast, for stories with distressing themes mentalizing was associated with the child’s previous attachment history and their risk for externalizing disorders (Hill et al., 2007, 2008).

For the present studies, children completed scripted story beginnings, themed with peer exclusion and victimization. Importantly, and unlike most exclusion research to date (see Wesselmann et al., 2015), the open-ended story-completion method offers subjects much latitude to express a range of post-exclusion responses. Specifically, we chose this measure as it enabled assessment of spontaneous prosocial and aggressive responses as well as children’s tendency to mentalize before and after exclusion. Though rarely, if ever, used in the context of an experimental task such as Cyberball, the story-completion approach is particularly appealing for use with young children, who may otherwise struggle to verbalize their thoughts (Emde et al., 2003).

## Study 1

Given the aforementioned links between affiliative and aggressive themes in children’s story-completions and parallel behaviors in various social contexts, it seemed plausible that exclusion would affect children’s play analogous to adults’ affiliative responses to exclusion (e.g., Maner et al., 2007). For typically developing children in Study 1, we predicted that compared to controls, excluded children would portray more affiliation between characters in stories. While studies report that social exclusion can elicit aggression (e.g., Twenge et al., 2001; Will et al., 2014), few if any child studies report such effects. Thus, we explored, but did not predict any effects of exclusion on aggression between characters.

Beyond affiliation and aggression, story-completion narratives are well-placed to examine post-exclusion attention to mental states. Thus we assessed the degree to which children treat story-characters as intentional agents (Hill et al., 2008). In line with enhanced post-exclusion social monitoring (Pickett and Gardner, 2005), we predicted that exclusion, compared to a control condition, would lead children to portray characters using more mental state language (MSL) and with more intentionality. Because social monitoring is thought to enhance reconnection (Molden and Maner, 2013), we also predicted that increases in mentalizing would mediate the effect of exclusion on affiliative story-themes.

Aside from testing our main hypotheses, in Study 1 we also employed character-specific codes to assess whether or not children selectively describe mental states of some story-characters and direct affiliation toward some characters over others (i.e., victims vs. perpetrators in the story). Social monitoring putatively helps to select good *and* weed out poor targets for affiliation (Pickett and Gardner, 2005). Accordingly, we predicted that a social exclusion condition would result in increased references to both the victim’s and perpetrators’ mental states compared to a control condition. Regarding affiliative portrayals, we expected that excluded children would favor victims over perpetrators, as victims should qualify as more promising sources of affiliation.

Finally, in selecting an appropriate control condition for Study 1, we were aware that inclusion cues can also promote both prosocial and antisocial responses (see Over and Carpenter, 2009a; Waytz, 2013) and that inclusion also activates fewer behavioral responses compared to exclusion (e.g., tattling; White et al., unpublished). Also, we aimed to ensure that children are responding to the perceived intentions of excluders. We therefore opted for an accidental exclusion control condition in which children were informed afterward that exclusion occurred due to a computer malfunction. This maps onto procedures in adult studies showing that affiliative responses are reliably elicited by rejecting departures compared to accidental departures (e.g., Maner et al., 2007). As a manipulation check for this control condition, we assessed whether or not children attributed more bad intentions to regular vs. accidental excluders on a puppet interview, after learning about the alleged computer malfunction.

### Method

#### Sample

Thirty-six 5-year-olds with a mean age of 68.26 months (*SD* = 2.43 months; 18 females) were recruited drawing on a database of families volunteering to participate in development studies. All subjects were native speakers. No ethnicity or SES data were available. Boys and girls were separately randomized to exclusion and accidental conditions. Ethical approval was obtained from Leipzig University’s institutional review board.

#### Procedure

Children initially completed a warm-up story themed with a Birthday party to acclimatize children to storytelling (Emde et al., 2003). After completing the story, they were informed that they could tell some more stories later. Next, children were furnished with a real-life glove and baseball, which they tossed back and forth with the experimenter. After a few throws, they were told that they would now play this game on the computer over the Internet. In the event that children were unfamiliar with the Internet, the experimenter explained that the Internet would allow them to play on the computer with two other children who were playing the game on a computer in different places, just like they were. Next, children played a first inclusion round of Cyberball, followed by an experimenter administering the first set of baseline story-stems. Then the child played a second experimental round of Cyberball during which they were initially included and then eventually either excluded or accidentally excluded (see section on Cyberball for manipulation details). Following either exclusion condition, a second set of story-stems was administered (stems counterbalanced to pre- and post-test). Puppet interviews were collected after administration of the second set of story-stems to assess attribution of bad intentions to co-players. Afterward, all children were over-included in Cyberball. An over-inclusion phase was deemed more suitable than debriefing for 5-year-olds in keeping with ethical guidelines for young children (see Thompson, 1990). Parents were fully debriefed after their child entered the lab, providing ample time to withdraw from the study before the child played Cyberball (no parents withdrew). Experimenters were blind to all research questions.

#### Measures

##### Cyberball (see **Figure 1**)

Cyberball is a computerized ball-toss game designed for adults (Williams et al., 2000) that was adapted for use with children (Crowley et al., 2010; see below). Subjects ostensibly played online with two other peers using a response pad. In fact, subjects were the only ones playing the game. Peers were computer-generated and their throws adhered to a pseudo-random event script. An initial inclusion period comprised of 30 trials, aimed to acclimatize children to the game interface. To help with comprehension of the task, an experimenter initially sat beside the child explaining the task and, if necessary, demonstrating the first throw before inviting children to try for themselves. After the eighth trial (third subject throw), experimenters complimented children on their performance and told them they had to do some paper work, taking a seat behind the child (while children completed the acclimatization round). The “acclimatization” round alternated between 9 “my turn” events (ball is thrown to participant), 9 “ball-toss” events (participant throws the ball) and 12 “not my turn” events (ball is passed between co-players).

**FIGURE 1:**
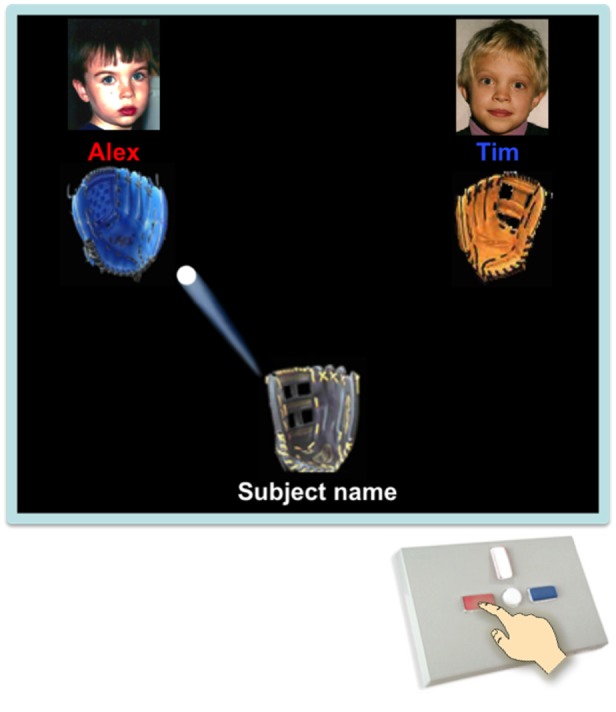
**Children played Cyberball ostensibly with two other children, whose photographs were displayed on the screen, using the red button to pass to the left player (name displayed in red) or the blue button to pass to the right player (name displayed in blue).** The children depicted in this figure are now adults and have provided their written consent for the publication of these identifiable images.

For the second experimental round of Cyberball, the experimenter immediately took a seat behind the child, pretending to work. The round was divided into a brief initial inclusion period of nine trials for all children (3 “my turn,” 3 “ball toss,” 3 “not my turn” events) seamlessly transitioning into exclusion (2 “my turn” events, 2 “throw events,” and 35 “not my turn” events). The exclusion and accidental conditions only differed in the two final screenshots appearing after the final ball-pass in the accidental condition. In the accidental condition, a first screenshot suggested that an error had occurred in red capital letters. Experimenters read this information out loud to children and terminated screenshots using the spacebar. The second screenshot showed a figure holding two disconnected ends of a red cable. To match this screenshot, response pads were connected to computers with a red sparkling USB cable and experimenters tampered with this cable when the second screenshot appeared. They also asked children if they had only received few balls, and told them that the other players could not toss the ball to them because the cable was disconnected. After the second set of story-stems and the puppet interview, all children played a third 38-trial over-inclusion round (16 “my turn,” 15 “ball toss,” and 6 “not my turn” events).

Crowley et al.’s (2010) version of Cyberball adds a number of child-friendly features. For example, a pre-recorded female narrator asks the child to pick their favorite from a selection of six baseball gloves before the game commences. For each throw the ball travels in one of many arcs from player to player (e.g., curved line), accompanied by a variety of swoosh sounds. Names and pictures of co-players were displayed above their gloves. Pictures of co-players were age and gender-matched, drawing on a picture bank of neutral child faces. Besides adding a new narrator to this version, we aimed to scaffold understanding of game controls. Thus, each time the subject caught the ball, names of co-players changed colors from white to red and blue to match the color of the respective button children had to press to throw the ball to that player (see **Figure 1**).

##### Story-stem administration

Following the MacArthur Story-Stem method (Bretherton and Oppenheim, 2003; Emde et al., 2003), standardized story-completions, enacted with Lego^®^ DUPLO^®^ figures, were used to elicit narratives from each child. Trained experimenters presented story beginnings to children following a standardized script before they asked children to “tell and show me what happens next”. Experimenters employed standardized prompts if children failed to address the problem presented in the stem. Before playing the acclimatization round of Cyberball, children completed a positively themed warm-up stem about a child’s birthday to check engagement and introduce all characters (Emde et al., 2003). Before and after the experimental Cyberball round children first completed a stem themed with peer-exclusion (“Sandbox,” “Snowman”) followed by a stem themed with peer-victimization (“Fight with a Friend,” “Favorite Chair”; Warren, 2003; Hill et al., 2007). Exclusion-themed stems were newly developed for this study (see Supplemental Material). We counterbalanced stems to baseline and experimental phases, so that each stem occurred equally often before and after exclusion. To standardize temporal gaps between stories and Cyberball, children were allowed to narrate stories for up to 3 min each.

##### Story-stem coding

All stories were transcribed and scored drawing on two different coding manuals and extensions of these systems (Robinson et al., 2002; Hill et al., 2009). All ratings were completed individually for each narrative from verbatim transcripts. Raters remained blind to the condition of subjects, other narratives of that child, order in which the stems were administered, and all other subject information. Raters received training from authors and/or experts of the respective coding systems. A second rater double-coded a random sample of 25% of stories (ICCs: 0.61 to 0.93).

Based on the first manual (Robinson et al., 2002) and in line with previous studies (von Klitzing et al., 2007), a composite of affiliative themes was formed for each story, involving empathy or helping (e.g., character puts band aid on other character), affection (e.g., characters hug), sharing (e.g., characters share items), reparation (e.g., character apologizes) and affiliation (e.g., characters play together) between characters. The presence of each theme was coded in a story and summed to a maximum score of five per story (*affiliation*). Each instance of affiliation was also coded in a new character-specific fashion. Two separate character-specific affiliative codes were derived by identifying the beneficiaries or recipients of each affiliative action, to create two separate affiliative codes. Affiliative actions were summed with the victimized party as recipients (*victim-directed affiliation*) and peers who perpetrated victimization as recipients (*perpetrator-directed affiliation*).

Based on a second coding manual (Hill et al., 2009), we coded the extent to which children globally portrayed characters as intentional agents (*intentionality*), i.e., as if they were goal-directed and had mental states (see Hill et al., 2007, 2008). Extending Hill et al. (2009) manual, we summed explicit intentional or mental state words children used to describe story-characters (e.g., “She *wants* to play with her in the snow.”) to create a score for *mental state language* (MSL) per story. To create a new set of character-specific scores we determined whether the child described a mental state of the victimized character (*victim-focused MSL*) or the characters perpetrating the victimization (*perpetrator-focused MSL*).

Additionally, we scored *aggression* between characters (Hill et al., 2009). Aggression assesses the extent to which children portray characters as acting aggressively toward one another, with higher scores reflecting more severe aggression. For example, verbal aggression usually scores in the lowest range (1–3), minor physical aggression in the intermediate range (4–6) while severe aggression resulting in injuries or even death rate in the high (7–9) or highest range (10–12), respectively.

To gain a more complete picture of narratives, we also scored story-quality (*coherence*) following a coding manual (Hill et al., 2009) and derived *word counts* from transcripts as a control a control variable using a standard software package (Pennebaker et al., 2007).

##### Preschool Ostracism Puppet Interview (POPI; White et al., unpublished)

We used a puppet interview protocol informed by the Berkeley Puppet Interview (Ablow and Measelle, 1993) to assess the extent to which children attributed bad intentions to their fellow players. Puppets claimed they had played the game as well and made opposing attributional statements regarding motives of their co-players (four items; “I think the other boys/ girls wanted to tease me” vs. “I don’t think the other boys/ girls wanted to tease me”). Interviews were videotaped and coded on seven-point scales (higher scores indicating stronger attribution of bad intentions; Cronbach’s α = 0.92). Over 25% of interviews were double-coded (*n* = 12; ICC = 1.00). Due to time-constraints, two children did not complete the interview.

#### Data-Analysis

We compared attribution of bad intentions by children in the exclusion and accidental conditions using analysis of variance (ANOVA). To compare conditions in regard to changes in global narrative codes from pre- to post-Cyberball on affiliation, MSL, aggression, intentionality, coherence, and word-count, we conducted a series of mixed-design ANOVAs, with time (pre- to post-Cyberball) as within-subject factor, and condition as between-subject factor. To analyze character-specific affiliation and MSL, we conducted two mixed-design ANOVAs, with time (pre- to post-Cyberball) and story-character (victim, perpetrator) as within-subject factors, and condition as between-subject factor. For all analyses, we averaged scores on peer-exclusion and peer-victimization stories before and after the manipulation after ensuring absence of Time by Condition by Story Type interactions. In a final step, we entered pre–post change in word count as a covariate in analyses of global narrative codes that yielded Condition × Time interactions, to ensure their independence of changes in story-length. The PROCESS macro (Hayes, 2013) was used to assess if changes in intentionality or MSL mediated effects of regular vs. accidental exclusion on changes in affiliative themes. Post-Cyberball affiliation and intentionality/ MSL scores were entered as independent and mediator variables, respectively, while pre-Cyberball scores functioned as covariates. We conducted ordinary least squares (OLSs) path analyses using 10,000 bootstrapping samples, a bias-corrected 95% confidence interval (CI), and omitted covariates to compute Preacher and Kelley’s (2011) κ^2^ as an effect size (small: 0.01 to 0.089, intermediate: 0.09 to 0.249, large: ≥0.25).

### Results

#### Manipulation check

An ANOVA revealed that excluded children attributed more bad intentions to their co-players, compared to children in the accidental condition, *F*(1,32) = 7.436, *p* = 0.010, $\eta_{p}^{2}$ = 0.189; *M_excl_* = 4.094; *SD_excl_* = 1.837; *M_accid_* = 2.625; *SD_accid_* = 1.284. This finding provides validity information regarding the accidental condition, supporting that preschoolers make distinctions between types of exclusion based on intentions of excluders.

#### Effects of exclusion on story-completions

**FIGURE 2:**
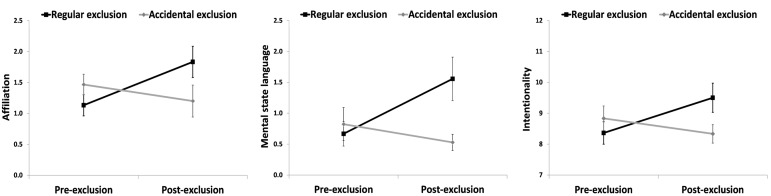
**Changes in children’s narrative portrayals of affiliation, mental state language, and intentionality in the exclusion compared to the accidental condition in Study 1**.

To test our hypotheses that exclusion would give rise to an increase in affiliation, intentionality, and MSL compared to the accidental condition, a series of 2 (Condition) by 2 (Time) repeated measures ANOVAs were performed (see **Table 1** for descriptives, *F*-values and effect sizes). No main effects of Condition or Time emerged for affiliation, intentionality, or MSL (*p*s > 0.12). Confirming our hypotheses, Condition × Time interactions were detected indicating greater increases after exclusion for affiliation (*p* < 0.001) as well as MSL (*p* = 0.004) and intentionality (*p* = 0.001) compared to the accidental condition (see **Figure 2**). Condition × Time Interaction effects on affiliation, MSL, and intentionality were robust to controlling for pre- to post-word count changes (*p*s < 0.014). The same analyses were conducted for coherence, aggression, and word count. Coherence yielded a main effect of time (*p* = 0.025), but neither an effect of condition (*p* = 0.652), nor a Condition × Time interaction (*p* = 0.593). No main effects or Condition × Time interactions emerged for word count (*p* = 0.131) or aggression (*p* = 0.626; see **Table 1**).

**Table 1:** Means and ANOVA results testing effect of condition (exclusion, accidental exclusion) on global codes in pre- and post-Cyberball doll-play narratives in Study 1.

	Mean narrative score		ANOVA (df = 1, 34)					
		
	Pre	Post	Condition (C)		Time (T)		C × T	
				
	M *(SD)*	M *(SD)*	*F*	$\eta_{p}^{2}$	*F*	$\eta_{p}^{2}$	*F*	$\eta_{p}^{2}$
Affiliation			0.09	0.003	2.56	0.070	15.07^∗∗∗^	0.307
Exclusion	0.94 (0.70)	1.61 (1.09)						
Accidental	1.33 (0.79)	1.06 (0.97)						
Aggression			0.10	0.003	3.41^†^	0.091	0.24	0.007
Exclusion	1.86 (0.98)	2.17 (1.70)						
Accidental	1.86 (1.00)	2.39 (1.12)						
Mental state language			2.09	0.058	2.27	0.063	9.52^∗∗^	0.219
Exclusion	0.67 (0.84)	1.56 (1.49)						
Accidental	0.83 (1.14)	0.53 (0.55)						
Intentionality			2.17	0.014	2.07	0.057	13.61^∗∗∗^	0.286
Exclusion	8.36 (1.54)	9.50 (1.99)						
Accidental	8.83 (1.70)	8.33 (1.27)						
Coherence			0.208	0.006	5.47^∗^	0.139	0.29	0.008
Exclusion	7.56 (1.68)	8.22 (1.99)						
Accidental	7.42 (1.95)	7.83 (1.86)						
Word count			0.463	0.013	1.65	0.046	2.393	0.066
Exclusion	46.44 (39.36)	56.06 (44.17)						
Accidental	43.86 (31.01)	42.97 (26.92)						


*^†^*p* < 0.10; ^∗^*p* ≤ 0.05; ^∗∗^*p* ≤ 0.01; ^∗∗∗^*p* ≤ 0.001.*

**FIGURE 3:**
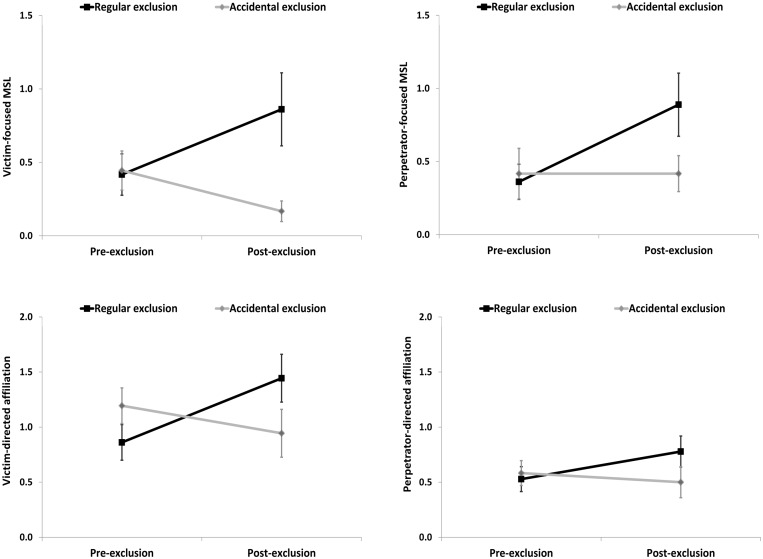
**Change in children’s narrative portrayals of victim- and perpetrator-focused MSL and victim- and perpetrator-directed affiliation and in the exclusion compared to the accidental condition in Study 1**.

To test our hypothesis that excluded children, but not controls, would preferentially direct affiliation toward the victim of the story, a 2 (Time) by 2 (Condition) by 2 (Character: victim or perpetrator) mixed-design ANOVA was performed. For affiliation, we detected a Condition × Time interaction, *F*(1,34) = 11.900, *p* = 0.002, $\eta_{p}^{2}$ = 0.259, which was further moderated by Condition × Time × Character interaction, *F*(1,34) = 5.100, *p* = 0.030, $\eta_{p}^{2}$ = 0.130. Two follow-up 2 (Time) by 2 (Condition) ANOVAs, revealed Condition × Time interactions for affiliation that was victim-directed (*p* = 0.001), but only at trend-level for affiliation that was perpetrator-directed (*p* = 0.057). This pattern of results suggested that excluded children increased victim-directed affiliation, but not perpetrator-directed affiliation compared to children in the accidental condition (see **Figure 3**, lower panels). For MSL, we also performed a 2 (Time) by 2 (Condition) by 2 (Character: victim or perpetrator) mixed-design ANOVA. Here, we detected a Condition × Time interaction, *F*(1,34) = 9.047, *p* = 0.005, $\eta_{p}^{2}$ = 0.210, but no evidence for a Condition × Time × Character interaction, *F*(1,34) = 0.468, *p* = 0.499, $\eta_{p}^{2}$ = 0.014. This pattern of results indicated that excluded children increased victim-focused and perpetrator-focused MSL to a comparable extent relative to children in the accidental condition (see **Figure 3**, upper panels).

From simple mediation models employing OLS path analysis, we found evidence that regular vs. accidental exclusion generated an increase in affiliation through their indirect effects on intentionality (CI for indirect effect: -0.416 to -0.017) as well as MSL (CI for indirect effect: -0.385 to -0.044). The mediation effects were medium to large for intentionality (κ^2^ = 0.201; CI = 0.053 to 0.395) and MSL (κ^2^ = 0.165; CI = 0.052 to 0.332).

## Study 2

In Study 2, we aimed to test the proposal that childhood anxiety may coincide with stress-induced deficits in mentalizing (e.g., Nolte et al., 2011). Accordingly, we predicted that children with ADs would exhibit a decline in depicting story-characters using MSL and intentionality after exclusion compared to controls. In this study, we thus exposed all children to regular exclusion and examined its effect as a function of anxiety. Concerning affiliative themes, we did not make specific predictions because the research is inconsistent, with some work suggesting that anxious children are highly motivated to be accepted by others (Banerjee, 2008), but other research indicating that individuals with (social) anxiety have trouble enacting reconnection behaviors after exclusion (Mallott et al., 2009). For this study, we also broadened our age-range as compared to Study 1. We did this, first, because we aimed to provide initial evidence that the patterns documented in Study 1 are not circumscribed to preschoolers, but also generalize to older children. Second, pragmatic reasons also played a role as the recruitment of clinically referred young children with diagnosed ADs also posed a challenge.

### Sample

Twenty clinically referred 4 to 8-year-olds with AD participated in this study prior to enrollment in a treatment-evaluation study (see Göttken et al., 2014). Following referral by a senior child psychologist of the outpatient services, presence of AD was independently established by a trained researcher using a diagnostic interview with the parent (see below). As a control group, 15 non-referred age- and gender-matched children were recruited via telephone from a group of volunteers for studies of child development. All children of the comparison group scored below the clinical cut-off of the emotional symptoms subscale of the Strengths and Difficulties Questionnaire (SDQ; Goodman, 1997; see below), which assesses anxiety and mood symptoms. The control group (hereafter referred to as non-anxious children or controls) was also comparable to the AD group in regard to years of parental schooling as well as rate of parental separation (see **Table 1**). All children in the AD group were recommended for enrollment in a treatment-evaluation study (see Göttken et al., 2014). Ethical approval was obtained from Leipzig University’s institutional review board.

### Procedure

All steps matched the regular exclusion condition of Study 1, with the following exceptions: AD children completed a puppet interview on their symptoms (not analyzed herein) prior to engaging in the procedure. To minimize the time-burden for AD children, the POPI was omitted after completion of the second set of story beginnings.

### Measures

#### Cyberball

The identical set-up was used as for the exclusion condition in Study 1.

#### Story-stem narratives

Administration (e.g., counterbalancing) and coding procedure of child narratives matched Study 2 in all regards, except the following: coding was limited to hypothesis-related dimensions of affiliation, aggression, coherence, intentionality, and MSL. A random sample of 20% of the present stories were double-coded by trained coders (ICCs: 0.66 to 0.86).

### Psychiatric Disorders and Symptoms

#### Preschool Age Psychiatric Assessment (PAPA)

The interviewer-based *Preschool Age Psychiatric Assessment* (PAPA; Egger and Angold, 2004) was administered to mothers of the AD group. The PAPA is a 2–3 h structured clinical interview to assess DSM-IV criteria of preschool and young school-age children below age 9 (Egger, 2012, personal communication). Across a 3-month primary period, mothers report frequency, duration and onset of child psychiatric symptoms to the interviewer. After entering all data into the electronic interview interface of the PAPA, algorithms designed by the developers of the PAPA and implementing DSM-IV criteria generate symptom scores and categorical diagnoses. The PAPA was translated and adapted between 2009 and 2010 by a research group at the University of Leipzig, assisted by the US PAPA authors. PAPA modules included in this study were: Oppositional Defiant Disorder (ODD), Conduct Disorder (CD), Depression (D), Social and Specific Phobia (SOP; SP), General Anxiety Disorder (GAD), and Separation Anxiety Disorder (SAD). A high degree of inter-rater reliability was established on primary diagnoses and subthreshold diagnoses (kappa coefficient = 0.92; range: 0.62 to 1.00; Göttken et al., 2014). The PAPA has shown good test-retest reliability and construct validity (Egger and Angold, 2006; Egger et al., 2006).

#### Strengths and Difficulties Questionnaire

All caregivers completed the 25-item Strengths and Difficulties Questionnaire (SDQ; Goodman, 1997) – a commonly used child-psychiatric screener that yields symptom scores for emotional symptoms (i.e., anxiety and mood symptoms), conduct problems, hyperactivity, and peer problems. Validity and adequate reliability for English and German versions were established in several studies (Goodman, 2001; Klein et al., 2013), for example, showing significant overlap between clinician-rated emotional disorders and parent-rated emotional symptoms (Becker et al., 2004). To screen the control group negative for anxiety symptoms, the Emotional symptoms subscale was checked to ensure that all controls scored below the clinical cut-off of 5, established within a representative German sample (Woerner et al., 2004).

### Verbal Competence

Receptive verbal ability was assessed using the picture-based Peabody Picture Vocabulary Test-Revised (PPVT-R; Dunn and Dunn, 1981) to ensure that groups were comparable in terms of verbal competence.

### Data-Analysis

First, to confirm successful matching, anxiety-disordered children and controls were compared on all demographic factors and verbal competence using χ^2^ and a series of one-way analyses of variance (ANOVA). For the main analyses, a series of two-way 2 (Time: Pre- vs. Post-exclusion) by 2 (Group: AD group vs. Controls) mixed-design analyses of variance (ANOVA) were conducted to assess group by time interactions on intentionality, MSL, coherence, aggression and affiliation.^2^ Significant interactions were followed up with separate one-way repeated measures ANOVAs in both groups to analyze whether effects of time (Time: Pre- vs. Post-exclusion) in the AD or the control group or both accounted for the results.

### Results

**FIGURE 4:**
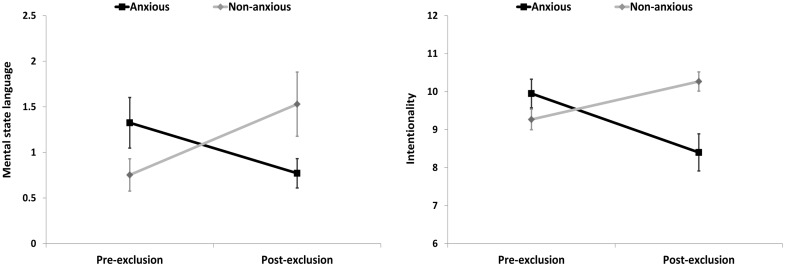
**Changes in anxious and non-anxious children’s narrative portrayals of MSL and intentionality in Study 2**.

Children with ADs were comparable to non-anxious controls on child age, gender, verbal competence, rate of parental separation, and parental education (all *p*s > 0.10; see **Table 2**). To compare AD children with controls on pre- to post-exclusion changes in narrative dimensions (prosociality, aggression, coherence, intentionality, MSL), a series of mixed-design ANOVAs were conducted (see **Table 3** for means, standard deviations, and test statistics). For intentionality and MSL, no main effects of group or time were observed, but, as predicted, an interaction between group and time emerged for intentionality (*p* < 0.001) and MSL (*p* < 0.006), showing that intentionality and MSL decreased from baseline to post-exclusion in the AD group, but increased for controls (see **Figure 4**). To check whether the interaction effect mainly derived from the decrease in the AD group or the increase among controls, a *post hoc* repeated measures ANOVA was conducted separately for each group with time as within-group variable. This revealed an increase in the non-anxious control group on intentionality, *F*(1,14) = 13.55, *p* = 0.002, $\eta_{p}^{2}$ = 0.492, and MSL, *F*(1,14) = 6.175, *p* = 0.026, $\eta_{p}^{2}$ = 0.306, as well as decrease in the AD group on intentionality, *F*(1,19) = 10.322, *p* = 0.005, $\eta_{p}^{2}$ = 0.352, and trend for a decrease on MSL, *F*(1,19) = 3.048, *p* = 0.097, $\eta_{p}^{2}$ = 0.138. Similarly, coherence also revealed a significant interaction effect (*p* < 0.001). Again, separate *post hoc* repeated measures ANOVAs were conducted for each group with time as within-group variable. This revealed both an increase in the control group, *F*(1,14) = 11.455, *p* = 0.004, $\eta_{p}^{2}$ = 0.450, as well as a decrease in the AD group, *F*(1,19) = 5.93, *p* = 0.022, $\eta_{p}^{2}$ = 0.246. No main effects of group or time, or interactions between time and group emerged for affiliation (*p*s > 0.23) and aggression (*p*s > 0.11).

**Table 2:** Demographic data of children with and without anxiety disorder in Study 2.

	Anxiety disorder (*n* = 20)	Non-anxious controls (*n* = 15)	AD vs. NAC	
			
Demographic data			Test-statistic	*p*
Mean child age in months *(SD)*	82.80 (15.41)	86.33 (13.52)	*F*(1,33) = 0.50	0.485
% females	50.00	46.67	χ^2^(1) = 1	0.845
% single parents	45.00	26.67	χ^2^(1) = 1.23	0.267
Parental education (Median)	High School Diploma	University Degree	*U*(33) = 78	0.107
Mean verbal score	75.80 (13.27)	81.55 (7.89)	*F*(1,33) = 2.22	0.146


**Table 3:** Means and ANOVA results testing effect of group (anxious, non-anxious) on global codes in pre- and post-Cyberball doll-play narratives in Study 2.

	Mean narrative score		ANOVA (df = 1, 33)					
		
	Pre	Post	Condition (C)		Time (T)		C × T	
				
	M *(SD)*	M *(SD)*	*F*	$\eta_{p}^{2}$	*F*	$\eta_{p}^{2}$	*F*	$\eta_{p}^{2}$
Affiliation			0.82	0.024	1.47	0.043	0.078	0.002
Anxious	1.38 (0.84)	1.50 (0.74)						
Non-anxious	1.60 (1.00)	1.80 (0.98)						
Aggression			2.05	0.059	1.60	0.046	2.59	0.073
Anxious	3.30 (2.63)	3.20 (2.51)						
Non-anxious	1.90 (1.00)	2.73 (1.27)						
Mental state language			0.12	0.003	0.24	0.007	8.52^∗∗^	0.205
Anxious	1.32 (1.24)	0.77 (0.72)						
Non-anxious	0.75 (0.68)	1.53 (1.36)						
Intentionality			1.63	0.047	0.82	0.024	17.69^∗∗∗^	0.349
Anxious	9.95 (1.69)	8.40 (2.19)						
Non-anxious	9.27 (1.05)	10.27 (0.98)						
Coherence			2.56	0.072	0.10	0.003	15.45^∗∗∗^	0.319
Anxious	8.43 (2.00)	7.40 (2.74)						
Non-anxious	8.33 (1.29)	9.53 (1.56)						
Word count			1.28	0.037	2.93^†^	0.082	0.133	0.004
Anxious	97.00 (61.72)	107.88 (87.20)						
Non-anxious	70.97 (44.58)	87.73 (44.58)						


*^†^*p* < 0.10; ^∗∗^*p* ≤ 0.01; ^∗∗∗^*p* ≤ 0.001.*

## Discussion

This research is the first to show that exclusion leads young children to shift how much they attend to others’ mental states and that the extent to which they do so depends on their level of anxiety. Thus, exclusion, but not accidental exclusion, led typically developing preschoolers to tell stories that portrayed characters as intentional agents, with more references to characters’ mental states, and increased affiliation between characters (Study 1). Conversely, young children with ADs were less likely to portray characters as intentional agents and made fewer references to story-characters’ mental states after exclusion compared to a non-anxious control group who showed similar increases on these dimensions as in the first study (Study 2).

Across Studies 1 and 2, we provide the field with first experimental data documenting young children’s systematic moment-to-moment fluctuations in attention to others’ mental states. During this crucial stage of development in understanding mental states, children already appear capable of flexibly increasing or decreasing mentalizing to meet the needs of a given situation. Indeed, exclusion may compel children to increase mentalizing, paving the way toward more effective reconnection (Pickett and Gardner, 2005), as suggested by the parallel increase in affiliative story-themes and their mediation by intentionality and MSL in Study 1. Moreover, considering the character-specific findings, children appear to monitor other minds broadly (victims and perpetrators alike), but direct their affiliative motivation specifically to those targets who are most open to cooperation (victims).^3^ Excluded children’s contemplation of the mental states of those around them may thus help them navigate toward target individuals who are most worthwhile to approach in order to restore a sense of connection. In turn, closely attending to a target’s mental states may also facilitate post-exclusion affiliative behaviors by the excluded party, given that genuinely prosocial and cooperative actions demand that the actor keeps the needs and goals of the recipient in mind (Tomasello, 2014). In that sense, excluded children may be thought of as adopting a “cooperative mindset.”

A distinct, but related interpretation of our data may suggest that exclusion prompted children to more strongly anthropomorphize story-characters in an attempt to cope with exclusion. Indeed, other studies have documented that exclusion or a dispositionally high need for inclusion leads individuals to anthropomorphize ambiguous or inanimate agents, thus augmenting the perception of social connection (Epley et al., 2008; Powers et al., 2014). Scholars have speculated that these patterns may assist excluded individuals in seeking solace in imaginary “parasocial” relationships or reflect adjustment of information-processing thresholds after exclusion to seek out new partners in more places (Knowles, 2013; Molden and Maner, 2013). We would suggest that this account complements the view that excluded children adopt a “cooperative mindset,” in that increased mentalizing post-exclusion may prepare children should opportunities for reconnection arise.

However, adopting a “cooperative mindset” does not appear to be a universal response to exclusion. Indeed, young children with ADs instead showed a decline in attending to mental states after exclusion. This deficit in mentalizing upon social threat therefore provides one potentially important reason why anxious children may have trouble applying their intact mentalizing skills to affectively charged social situations (see Banerjee, 2008). Excessive negative arousal, typical of childhood anxiety, may interfere with controlled mentalizing, potentially resulting in a more automatic mode of mentalizing after exclusion, coinciding with reflexive assumptions about others’ internal states (Fonagy and Luyten, 2009).

Notably, we recently reported neural data suggesting that insecure attachment strategies lead children to respond to the Cyberball paradigm with more excessive and enduring negative expectations regarding re-inclusion than securely attached children (White et al., 2012, 2013). The present anxiety-related drop in mentalizing could set the stage for an over-extension of these negative expectations to other encounters after exclusion. Specifically, anxious children might effectively be making unjustified, reflexive, and sweeping assumptions about the mental attitudes of others toward themselves (automatic mentalization) that promotes generalization of their own negative views of themselves, others, and the world (“Nobody will ever let me back in”). Inasmuch as reduced mentalizing may then, in turn, impede affiliation after exclusion, it may partly explain why childhood anxiety is associated with increased risk for peer rejection in many studies (e.g., Perren et al., 2006; von Klitzing et al., 2014). Indeed, given that most individuals get exposed to exclusion at some point or another (Nezlek et al., 2012) – perhaps especially so in early childhood when children are less socially skilled and exclusion may even occur accidentally (Monks, 2011) – much may depend on the capacity to recover from exclusion once it has transpired.

### Limitations and Future Directions

First, it may seem surprising that anxious children did not also evidence diminished affiliative themes in their story-completions in Study 2. However, scholars frequently caution against equating portrayals in story-completions with the actual experiences they denote (e.g., Bretherton and Oppenheim, 2003). The exclusion-induced increase in affiliative portrayals in Study 1 may thus potentially signify a behavioral disposition of the excluded child or a wish for such behavior from others, rather than the behavior or experience itself. Perhaps anxious children preserve their wish and motivation to be accepted by others, despite a failure to act accordingly to reach this goal (Banerjee, 2008), which would reconcile our findings with data showing diminished post-exclusion reconnection behaviors among socially anxious adults (Mallott et al., 2009). Given that we have shown that social exclusion impacts what children “think about,” future work may examine how attention to mental states relates to what they actually do, for instance, if given an opportunity to “reunite” (White et al., 2013) or if aggressive options are available (Warburton et al., 2006).

Second, our data also raise important questions regarding the exclusion-specificity of the observed changes in mentalizing for typically developing and anxious children. To draw conclusions on this issue, we would need to compare effects of various types of stressors (e.g., negative pictures, tackling unsolvable tasks, losing a game). However, we speculate that other social-evaluative stressors (e.g., giving a presentation to an audience) would also generate similar results. Indeed, even non-social threat may sometimes kindle an affiliative motivation (Schachter, 1959), and may therefore, by extension, also lead to elevated mentalizing among healthy individuals. Future research could attempt to disentangle the effects of arousal and affiliative motivation in different populations.

Third, in a related vein, future research should also aim to specify the dispositional factors that influence context-dependent shifts in mentalizing. Indeed, in other work using the story-completion method, conduct disorders and externalizing symptoms have also been associated with reduced portrayals of characters as intentional agents, but only in stories with distressing themes (Hill et al., 2007, 2008). In keeping with recent proposals, stress-induced mentalizing deficits may therefore reflect a transdiagnostic vulnerability to mental disorder, rather than a vulnerability specific to anxiety (see Fonagy et al., 2016). Future work could examine children with other clinical problems that promote high arousal under challenge (e.g., aggression), likely impeding children in bouncing back from rejection.

Fourth, it is also noteworthy that unlike some behavioral data in adults (Twenge et al., 2001), we did not observe any increases in aggressive story-themes in our data either among typical or anxious young children. Interestingly, this corresponds to a finding in our previous study, showing that preschoolers in contrast to adults do not feel threatened in their subjective sense of control by exclusion (White et al., unpublished). Notably, control-threat has been identified as the single-most important mediator of aggressive responses to exclusion, as excluded individuals act aggressively to regain a sense of agency and influence over events (Gerber and Wheeler, 2009). Potentially, during this early period when children are still gaining familiarity with peer interactions and may show greater generosity than at later stages (Fehr et al., 2008), peer exclusion may serve as a stronger suppressant of aggression than at later stages (Barner-Barry, 1986). More generally, this null-finding additionally strengthens our conclusion that the increases in mentalizing observed here primarily occurred in the context of a motivation to reconnect. Yet, a sample which included dispositionally aggressive children may potentially yield increases in aggressive story-themes.

Fifth, in this study we used a story-completion measure to assess the degree to which children engage in mentalizing following exclusion. However, it is conceivable that other measures of mentalizing, such as standard false belief tasks that tap into the capacity to infer beliefs that contrast with the child’s own knowledge (Wellman, 2014), may yield divergent results. For a more complete picture, researchers should also aim to administer such tasks before and after exclusion in future studies.

Sixth, future work should also assess healthy and anxious children’s responses to inclusion conditions. For the present study, an inclusion condition was primarily deemed less appropriate, given that previous studies document that inclusion cues may also promote cooperation and trust (Over and Carpenter, 2009a; Hillebrandt et al., 2011). Therefore, inclusion may prove suboptimal as a control condition to examine reconnection responses to exclusion. However, inclusion responses may be of interest in their own right.

Finally, a set of alternative interpretations also deserve attention. Thus, it might be suggested that children merely ponder mental states of others after exclusion because they are wondering why they were excluded. Indeed, Cyberball is a causally ambiguous task (Williams and Zadro, 2005), i.e., participants are not informed why their co-players stopped passing them the ball. However, if increased mentalizing merely reflected a wish to understand the reasons for exclusion in Cyberall, excluded children would be expected to focus their attention more narrowly on mental states of perpetrators in their stories. Yet, we did not find evidence for this using character-specific scores in Study 1. A second account might suggest that Cyberball gives children a firsthand experience of exclusion that leads to a better understanding of mental states of story-characters facing similar situations. However, if this were the sole explanation, excluded children might primarily be expected to better understand mental states of the story-victim. Instead, we observed an increase in mentalizing in relation to victims *and* perpetrators. Notably, we are not claiming that neither of these social-cognitive processes operate after exclusion. Rather, we are suggesting that they are unlikely to fully explain our pattern of findings. Indeed, neither of these *lean* interpretations of our data are easily reconciled with the fact that intentionality and MSL mediated the effect of exclusion on affiliative story-themes in Study 1, suggesting that mentalizing in this context provides a means for reconnection and that young children may already flexibly adapt their level of mentalizing to match their affiliative goals.

## Conclusion

A developmental theory of mental state understanding is incomplete as long as we know relatively little about the circumstances and dispositions that determine the extent to which children actually use this competence or not. Our findings show that social exclusion offers an important stimulus for the usage of mentalizing from preschool age onward. As excluded children weigh the benefits of reconnection (promotion) against the cost of potential further rejection (prevention; Molden and Maner, 2013), attending to others’ mental states may provide a useful “mental reconnection tool” to vigilantly filter, approach, and re-engage with potential social partners. However, this “mental reconnection tool” may not be readily available to all children facing social exclusion. Thus, we showed that children with ADs exhibit a drop in mentalizing following exclusion. Given a general model of mentalization and regulation of negative affect (Fonagy et al., 2002), it is likely that the process of impaired mentalizing under the social challenge of exclusion reflects a transdiagnostic vulnerability factor that more broadly lies at the core of developmental psychopathology.

## Ethic Statement

Informed written consent was collected from all parents and all children also orally assented to the study. Four to 8-year-olds participated in a computerized ball-toss game during which they were eventually excluded. At the end of the procedure, all children were over-included in the game by a new set of children to dispel any potential negative emotions. An over-inclusion phase was deemed more suitable than debriefing for this age group in keeping with ethical guidelines for young children (Thompson, 1990). Parents were fully debriefed before the experimental procedure, providing ample time to withdraw from the study before the child played the ball-game (no parents withdrew).

## Author Contributions

Writing and revision of manuscript: LW, MC, AK, KvK, AG, YO, JH, HO, and PF; study design: LW, AK, KvK, MC, AG, YO, and PF; data-collection: LW, AK, and AG; and data analysis: LW, AK, AG, and YO.

## Conflict of Interest Statement

The authors declare that the research was conducted in the absence of any commercial or financial relationships that could be construed as a potential conflict of interest.

## Footnotes

1 Various dimensions of mentalizing have been operationalized (see Luyten and Fonagy, 2014, for an overview). Story-stem based measures primarily focus on the child’s tendency to attribute cognitive-affective mental states to others (i.e., story-characters) starting from the portrayals in the story-beginning. Notably, unlike standard false belief tasks (e.g., Wimmer and Perner, 1983), story-stem based assessments focus on the child’s spontaneous usage of mental state attribution rather than the accuracy of these attributions.

2 Including story-type in a three-way 2 (Time: Baseline vs. Post-exclusion) by 2 (Group: AD group vs. Controls) by 2 (Story Type: exclusion vs. peer-conflict) mixed-design ANOVA, yielded no evidence of a three-way interaction. Therefore, as in Study 1, we collapsed children’s scores across stories (i.e., using mean scores at baseline and post-exclusion).

3 The post-exclusion increase in victim-directed affiliation may also reflect an “attraction” to story-characters who share the subject’s plight (i.e., victimization), resembling classic findings reporting that subjects expecting a novel threat preferred to wait with similarly threatened others, rather than others in a dissimilar situation (Schachter, 1959; Gump and Kulik, 1997). Potentially other excluded parties may afford especially promising targets for reconnection, as they may share the subject’s desire to reconnect, given their equally excluded state.
