# Supplementary material for: Putting Ostracism into Perspective: Young Children Tell More Mentalistic Stories after Exclusion, But Not When Anxious
Source: Front Psychol. 2016 Dec 22;7:1926. doi: 10.3389/fpsyg.2016.01926 (PMC5177662; doi:10.3389/fpsyg.2016.01926)
Supplement: Supplementary file 1 [file Data_Sheet_1.DOCX]

**Supplemental material**

*Peer-exclusion story-stems*

Administration notes:

- experimenter portrays actions of figures using gender-matched Duplo dolls
- use gender-matched names (Susan = George, Laura = David, Katie = Robert*)*

“Sandbox” (female version)

Laura and Katie are playing on the playground with the ball. Susan is sitting over here in the sandbox near her parents, playing in the sand. But she would really like to play with her friends Laura and Katie. So she goes over to them. But when Katie sees Susan coming, she whispers into Laura’s ear.

Katie: “Oh no, look who’s coming… I don’t want to play with Susan.”

Then Susan asks:

Susan: “Can I play with you?”

Show and tell me what happens next.

“Snowman” (female version)

Now it is winter. Susan is really excited about playing with Laura in the snow. So she goes over to Laura and knocks on her door. When Laura opens up, Susan asks her:

Susan: “Can we go outside and play together?”

Laura: “Uhm. I haven’t got time today.”

Susan *(in a sad voice)*: “Oh. That’s a shame.”

So Susan goes back home to her parents and looks out of the window … and look what she sees there: Laura and Katie building a big snowman together outside on the lawn.

Show and tell me what happens next.
